# Change in the Interstitial Cells of Cajal and nNOS Positive Neuronal Cells with Aging in the Stomach of F344 Rats

**DOI:** 10.1371/journal.pone.0169113

**Published:** 2017-01-03

**Authors:** Yong Hwan Kwon, Nayoung Kim, Ryoung Hee Nam, Ji Hyun Park, Sun Min Lee, Sung Kook Kim, Hye Seung Lee, Yong Sung Kim, Dong Ho Lee

**Affiliations:** 1 Departments of Internal Medicine, Seoul National University Bundang Hospital, Seongnam, Korea; 2 Department of Internal Medicine, Kyungpook National University Hospital, Daegu, Korea; 3 Department of Internal Medicine and Liver Research Institute, Seoul National University College of Medicine, Seoul, Korea; 4 Departments of Pathology, Seoul National University Bundang Hospital, Seongnam, Korea; 5 Department of Gastroenterology and Digestive Disease Research Institute, Wonkwang University School of Medicine, Iksan, Korea; University of Nevada School of Medicine, UNITED STATES

## Abstract

The gastric accommodation reflex is an important mechanism in gastric physiology. However, the aging-associated structural and functional changes in gastric relaxation have not yet been established. Thus, we evaluated the molecular changes of interstitial cell of Cajal (ICC) and neuronal nitric oxide synthase (nNOS) and the function changes in the corpus of F344 rats at different ages (6-, 31-, 74-wk and 2-yr). The proportion of the c-Kit-positive area in the submucosal border (SMB) and myenteric plexus (MP) layer was significantly lower in the older rats, as indicated by immunohistochemistry. The density of the nNOS-positive immunoreactive area also decreased with age in the SMB, circular muscle (CM), and MP. Similarly, the percent of nNOS-positive neuronal cells per total neuronal cells and the proportion of nNOS immunoreactive area of MP also decreased in aged rats. In addition, the mRNA and protein expression of c-Kit and nNOS significantly decreased with age. Expression of stem cell factor (*SCF*) and the pan-neuronal marker *PGP 9*.*5* mRNA was significantly lower in the older rats than in the younger rats. Barostat studies showed no difference depending on age. Instead, the change of volume was significantly decreased by L-NG63-nitroarginine methyl ester in the 2-yr-old rats compared with the 6-wk-old rats (*P* = 0.003). Taken together, the quantitative and molecular nNOS changes in the stomach might play a role in the decrease of gastric accommodation with age.

## Introduction

Gastric accommodation is defined as the decrease in gastric tone and increase in compliance after the ingestion of meals [1]. Abnormality in this mechanism may explain the pathogenesis of postprandial fullness or early satiation [2]. Several studies reported that non-adrenergic, non-cholinergic (NANC) nerves are related to the inhibition of gastrointestinal(GI) smooth muscle [3]. Among these neurotransmitters, nitiric oxide (NO) has been considered as the important inhibitory neurotransmitter in GI tract [4–6]. Other neurotransmitters e.g., 5-hydroxytryptamine (5-HT) [7, 8], adenosine 50-triphosphate (ATP), and vasointestinal polypeptide (VIP) are also involved in gastric relaxation as co-transmitters of the enteric inhibitory neurons with NO [9]. Previous studies revealed that the aging process has been associated with gastric motility, such as a decrease in the relaxation of the stomach [10]. This change may induce an early satiation in elderly people [10, 11]. As a possible explanation of this change in elderly people, there has been a report that enteric neurodegeneration is related to the loss of excitatory cholinergic neurons [12]. In contrast, nitrergic myenteric neurons have been reported to be selectively spared during aging [13–15]. For instance, Philipps *et al*. suggested that age-related cell loss in the myenteric plexus (MP) did not occur in nitrergic neurons but occurred exclusively in the cholinergic subpopulation of enteric neurons in Fischer 344 rats [11, 14]. However, our group reported a decrease of nitrergic neurons in the colon muscle [16] and gastric mucosa of aged F344 rats [17]. Chapman also suggested that NO plays an important role in the mechanism of satiety, and the decline in the compliance might be caused by decreased production of NO with age [11, 18]. Another quantitative study reported a significant decrease in the number of neurons in the MP with advanced age [19].

In addition to NO, interstitial cells of Cajal (ICC) play an important role in gastrointestinal motility. ICC generate a rhythmic pacemaker current, which manifests itself as slow waves in the membrane potential of smooth muscle cells, resulting in rhythmic bowel contractions [20, 21]. However, the role of ICC in the transmission of inhibitory signals from enteric neurons to smooth muscle cells is highly controversial [11, 21]. Recently, Sanders *et al*. revealed that the inhibitory responses were absent or reduced when ICC-IM were partially or completely lost, providing evidence for the role of ICC-IM in mediating inhibitory neurotransmission in the gastric fundus of W/W^V^ mice [22]. Furthermore, decrease or loss of ICC in the human gut was frequently observed in the diabetic gastroenteropathy [23] or slow transit constipation [24, 25]. ICC express the proto-oncogene *c-Kit* [26, 27] and stem cell factor (SCF) which is a natural ligand of Kit [28], related to the development and maintenance of ICC [29]. In addition, up-regulation of the enzyme heme oxygenase-1 (HO-1), an important player in the cellular defense mechanism against oxidative stress [30, 31], and its product, carbon monoxide, has been reported to protect ICC from oxidative stress in diabetic models [32, 33]. These results suggest that HO-1, in addition to ICC, another important component in regulating gastric accommodation in older animals. However, in the role of nNOS and ICC of the rectoanal inhibitory reflex (RAIR) and electrical field stimulation (EFS)-induced internal anal sphincter relaxation, nNOS was the major mediator but ICC were not critical for the RAIR [34].

From this background, we aimed to elucidate the morphological and molecular changes in nNOS and ICC with SCF and HO-1 that could be involved in the gastric accommodation of the corpus in 6-, 31-, 74-wk and 2-yr old F344 rats, (which are equivalent to 5, 30, 60 and 80 years of human age, respectively). In addition, the function of corpus was evaluated using a gastric barostat at these four ages of rats.

## Materials and Methods

### Animals

Specific pathogen-free, male, F344 rats (four different age groups: 6-, 31-, and 74- wk- and 2 yr-old) were used (Orient Co. Ltd., Seoul, Korea). The animals were housed in a cage maintained at 23°C with 12:12-hour light-dark cycles and specific pathogen-free conditions. They were allowed unrestricted intake of food and water. This study was carried out in strict accordance with the recommendations in the Guide for the Care and Use of Laboratory Animals of South Korea. The protocol was approved by the Committee on the Ethics of Animal Experiments of the Institutional Animal Care and Use Committee (IACUC) of Seoul National University Bundang Hospital (BA1403-148/012-02). All experiments were performed between 9:00 AM and 6:00 PM. All rats were euthanized by carbon dioxide following experiments as approved by our IACUC Animal Care and Use Protocol.

### Tissue preparation and immunohistochemistry for c-Kit and nNOS

**Fig 1:**
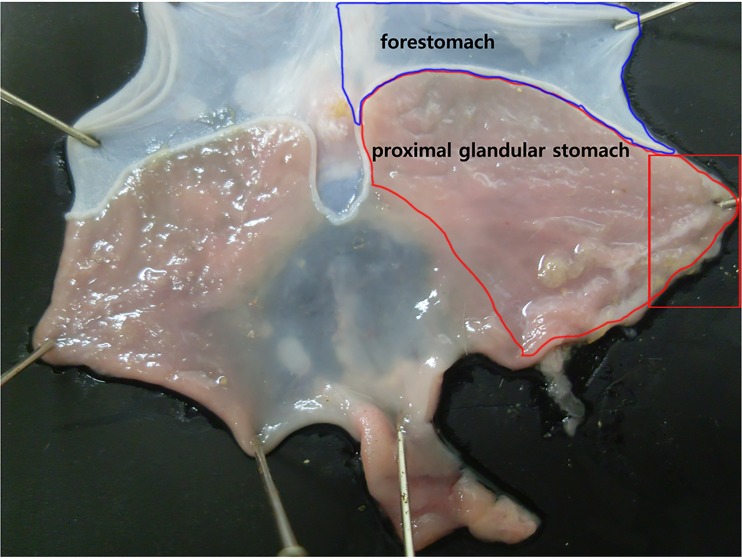
Diagram of rat stomach. The stomach of the rat is divided into the forestomach (pars proventricularis) and glandular stomach (pars glandularis). For this experiment, the proximal glandular stomach of F344 rat was used after removing forestomach. Stomach was opened along the greater curvature and about a one-centimeter length of proximal glandular stomach (corpus) was used for immunohistochemistry (rectangular box in the right side).

Histological analysis of tissue was performed by following the methods of previous reports [16, 17]. Briefly, a one-centimeter length of the proximal glandular stomach (corpus) of rats (Fig 1), not including forestomach, was obtained and fixed in 10% buffered formalin for histology. The specimens were embedded in paraffin, sectioned perpendicularly to the lumen (section thickness, 4 μm) and stained with hematoxylin and eosin (H&E). One H&E-stained slide per rat (n = 6 for each age group) and two fields per slide were randomly selected and checked for whether there was any difference of muscle area depending on age.

Immunohistochemistry was performed following the methods of the previous report [16]. The sections were incubated with the following primary antibody: anti-c-Kit antibody (dilution 1:100; polyclonal rabbit anti-human CD117, DAKO, Glostrup, Denmark) and anti-nNOS antibody (dilution 1:500; AB5380 Chemicon Millipore Corporation, Billerica, MA, USA) after deactivation of endogenous peroxidase with 3% hydrogen peroxide and blocking of nonspecific binding sites. The immunostaining was performed using an automatic immunostainer (BenchMark XT, Ventana Medical Systems, Inc., Tucson, AZ, USA) according to the manufacturer’s instructions. An UltraView Universal DAB detection Kit (Ventana Medical Systems) was used for the secondary antibody. The negative control for IHC was performed without primary antibodies. The immunostained tissues were examined under a light microscope (Carl Zeiss, Jena, Germany) linked to a computer-assisted image analysis system (AxioVision Rel.4.8; Carl Zeiss). Two immunostained slides per each rat (n = 6 for each age group) were prepared, and four to five fields per slide were randomly selected to obtain micrographs at x200. The micrograph was divided into four anatomic regions; submucosal border (SMB); circular muscle (CM); MP and longitudinal muscle (LM) regions [35] using Adobe Photoshop ver. 7.0 (Adobe systems; Mountain View, CA, USA). Finally, quantitative assessment of the c-Kit and nNOS immunoreactivity was performed using the Image-Pro® Plus analysis system (Media Cybernetics, Inc., San Diego, CA, USA), and measurements were expressed as the proportion of immunoreactive area (% of total area). Mast cells, known to express c-Kit, were excluded by their round or oval shape and lack of processes [23, 27, 36]. Ganglia in the myenteric plexus were micrographed at x1,000. The number and proportion of myenteric neurons, which include nuclei, were enumerated. All morphometry experiments were performed blinded to the identity of the samples.

### Real-time PCR for *c-Kit*, *SCF*, *HO-1* and *nNOS*

*c-Kit*, *SCF* and *nNOS* mRNA levels were measured by real-time PCR according to the method as described in detail previously[16]. Briefly, RNA was extracted from the corpus muscle tissues (Fig 1) devoid of the mucosa, submucosa and preferably serosa using an RNeasy Plus Mini Kit (Qiagen, Valencia, CA, USA) according to the manufacturer’s instructions. RNA samples were diluted to a final concentration of 0.5 mg/mL in RNase-free water and stored at -80°C until use. Synthesis of the cDNA was performed with 1 mg of total RNA with M-MLV reverse transcription reagents (Invitrogen, Carlsbad, CA, USA). The 20 μL reverse transcription reaction consisted of 4 μL of first-strand buffer, 500 mM deoxynucleoside triphosphate mixture, 2.5 mM oligo (dT) 12–18 primer, 0.4 U/mL ribonuclease inhibitor, and 1.25 U/mL Moloney murine leukemia virus 152 reverse transcriptase (Invitrogen). The thermal cycling parameters for the reverse transcription were 10 minutes at 65°C, 50 minutes at 37°C and 15 minutes at 70°C. Real-time PCR amplification and determination were performed using SYBR Premix Ex TaqTM (Takara Bio, Shiga, Japan) according to the manufacturer's protocols. The following primers were used: *c-Kit* forward, TTC CTG TGA CAG CTC AAA CG; *c-Kit* reverse, AGC AAA TCT TCC AGG TCC AG; *SCF* forward, CAA AAC TGG CGA ATC TT; *SCF* reverse, GCC ACG AGG TCA TCC ACT AT; *HO-1* forward, AAG AGG CTA AGA CCG CCT TC; *HO-1*, reverse, GCA TAA ATT CCC ACT GCC AC; *nNOS* forward, CTA CAA GGT CCG ATT CAA CAG; *nNOS* reverse, CCC ACA CAG AAG ACA TCA CAG; *GAPDH* forward, AGG TGA AGG TCG GAG TCA; and *GAPDH* reverse, GGT CAT TGA TGG CAA. The *GAPDH* gene was used as an endogenous reference as a control for expression that was independent of sample-to-sample variability. The amplification protocol consisted of an initial denaturation step at 95°C for 10 seconds, followed by 40 cycles of denaturation for 5 seconds at 95°C and annealing/extension of 33 seconds at 55°C. The relative expression levels of target genes were normalized by dividing the target Ct values by the endogenous Ct values. All equipment was purchased from Applied Biosystems and used according to their protocols. RNA-free water was used in real-time PCR for the no-template control (NTC). After amplification, we performed melting curve analysis using ABI PRISM® 7000 Sequence Detection System software (Applied Biosystems).

### Western blotting for c-Kit and nNOS

The corpus muscle tissue devoid of mucosa, submucosa and preferably serosa was homogenized with lysis buffer containing 25 mM Tris-HCL (pH 7.4), EGTA (1 mM), DTT (1 mM), leupeptin (10 μg/mL), aprotinin (10 μg/mL), PMSF (1 mM), and Triton X-100 (0.1%), as described in detail previously [18]. Briefly, the proteins (100 μg for each sample) were separated by SDS-PAGE (8% wt/wt gel) and transferred to PVDF membranes. All procedures were performed in Tris buffer (40 mM, pH 7.55) containing 0.3 M NaCl and 0.3% Tween 20. The membranes were then blocked with dried milk (5% wt/vol) and subsequently incubated with antibodies for c-Kit (1:100; rabbit polyclonal antibody, Santa Cruz Biotechnology, Santa Cruz, CA, USA), nNOS (1:500; mouse monoclonal IgG2a antibody, BD Biosciences, San Diego, CA, USA) and β-actin (1:1000; rabbit polyclonal antibody, BioVision, Milpitas, CA, USA) at 4°C overnight. The blots were incubated with secondary antibody (rabbit polyclonal antibody, Santa Cruz Biotechnology for c-Kit (dilution 1:500) and β-actin (dilution 1:1000) and mouse polyclonal antibody (1:1000; Santa Cruz Biotechnology) for nNOS, and an imaging analyzer was used to measure the band densities. For c-Kit immunoblot, the optical densities of the mature (145 kDa) forms were combined into one parameter for the analysis process using densitometry [37].

### Gastric barostat study and evaluation of gastric relaxation as a function of age

**Fig 2:**
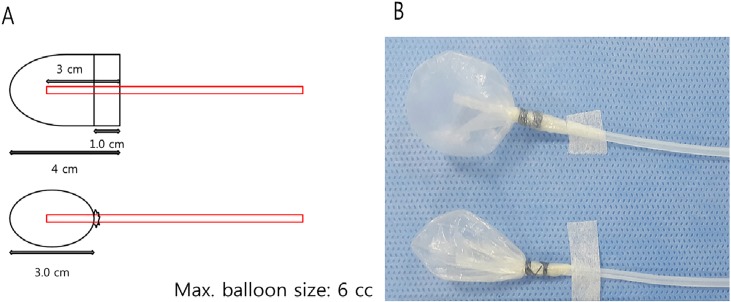
The intra-gastric balloon. (A) A polyethylene bag was made by using a 3^rd^ finger-tip of poly-glove (Hanjin, Seoul, Korea). Maximal diameter is 3 cm. (B) The maximum volume of balloon was 6 mL and air was injected into the balloon from one of the balloon tubes with the other side balloon tube closed to allow placement of the balloon within the stomach, after which the balloon tubes were immediately opened to the air.

**Fig 3:**
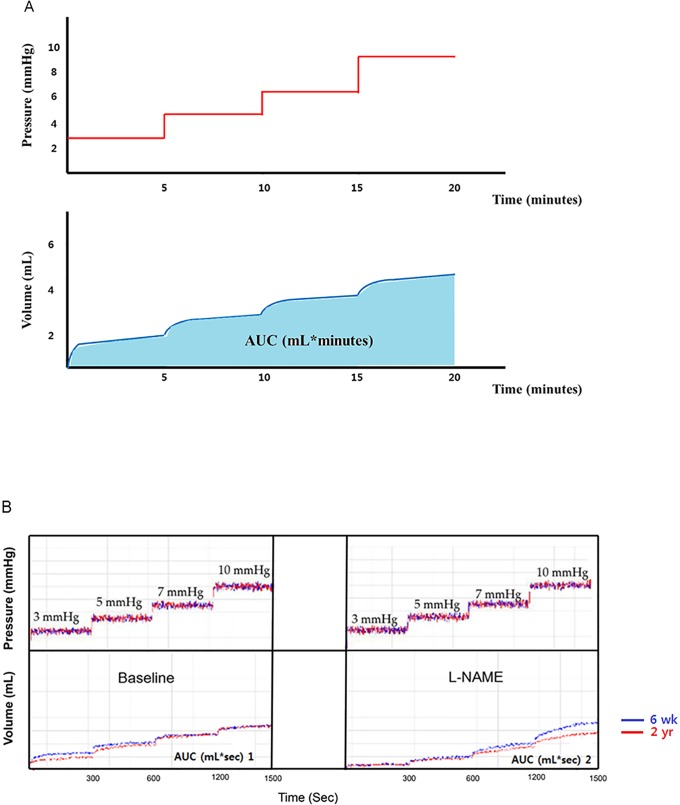
Measurement of the change in gastric accommodation. (A) The pressure-dependent volume curve obtained by applying 3, 5, 7, and 10 mmHg at 5 minute intervals was measured in each aged rat group. The change of volume and area under curve (AUC) of the pressure-dependent volume was measured in each group to obtain the pressure-related volume (mL) * time (sec) in the tonic phase. (B) To analyze the gastric accommodation in each aged rat group, we compared with the pressure associated volume change and baseline AUC (mL*sec) of the aged rat groups. Afterwards, we compared the change in AUC (Δ AUC: AUC 2—AUC 1) after administering L-NAME to analyze the response of nNOS activity with age indirectly. The blue and red lines in the graph represent the pressure and volume curves of the 6-wk- and 2-yr-old rats. L-NAME, L-NG-nitroarginine methylester.

The rats fasted for 24 hours prior to the experiments but were allowed access to water. The animals were anesthetized by a Zoletil (Virbac, France) and Rompun (Bayer Korea, South Korea) mixture. A pair of polyvinyl tubes attached to a polyethylene bag (Fig 2A & 2B) was introduced through the mouth and passed into the stomach [38]. In a separate group of fasted rats, an electronic barostat (G & J Electronics Inc, Willowdale, Ontario, Canada) was applied to assess gastric accommodation. For full dilatation of intragastric balloon, the initial distention pressure of 15 mm Hg was maintained for one min, there was a 15 min resting period with deflation of balloon, and then, the pressure was increased in a stepwise fashion, without intervening deflation, to 3, 5, 7, and 10 mmHg in 5 min intervals (tonic phase). After this cycle, the pressure was decreased to the minimum distention pressure and maintained for another 20 min. The gastric volume produced at the minimum distention pressure during the first 10 min is referred to as the baseline. After a resting period, L-NG-nitroarginine methyl ester (L-NAME) was dissolved in distilled water for injection and administered intravenously to the rat tail vein at a dose of 30 mg/kg (0.1 mL/100 g body weight) 10 min before the barostat study for gastric relaxation [38]. For the measurement of gastric relaxation, the pressure associated volume change and the area under the curve (AUC) of the pressure-dependent volume curve of age group was compared (Fig 3A). In addition, the response to L-NAME was measured by comparing the change in AUC (Δ AUC: AUC 2 –AUC 1) between the L-NAME-induced pressure-dependent volume curve (AUC 2, mL*sec) and the basal pressure-dependent volume curve (AUC 1, mL*sec) in the four F344 rat groups. Fig 3B shows the comparison of pressure dependent volume curve between 6-wk- and 2-yr-old F344 rats.

### Statistical analyses

All continuous variables were expressed as mean ± standard deviation (SD). Multiple comparisons among different aged rat group were performed using the Tukey test and Wilcoxon rank sum test, respectively. The change of intrabag volume from baseline and areas under the curve (AUC) were calculated and compared using ANOVA and Tukey’s posttest. *P* values < 0.05 were considered statistically significant. All statistical analyses were performed using SPSS software (version 20.0; SPSS Inc., Chicago, IL, USA).

## Results

### Influence of aging on the c-Kit-positive area in the rat corpus

**Fig 4:**
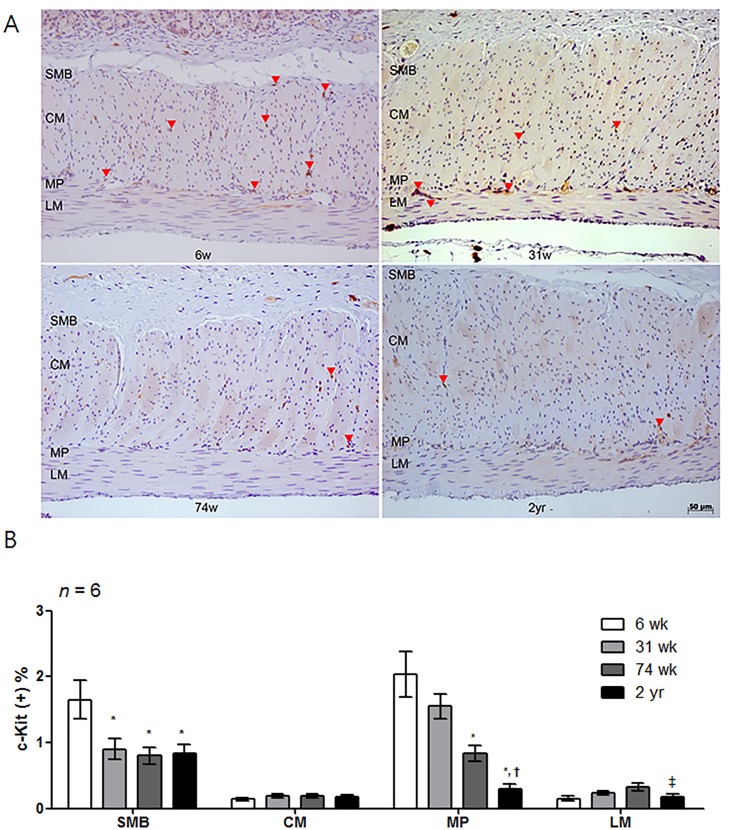
Analysis of c-Kit immunohistochemistry. (A) Photomicrograph of c-Kit immunostaining of the proximal rat stomach. Arrows indicate the c-Kit immunoreactive cells (x200 magnification). (B) Comparison of the proportion of the c-Kit immunoreactive area of the SMB, MP, CM and LM in 6-, 31-, 74-wk- and 2-yr-old rats (*n* = 6 per group). The proportion of the c-Kit immunoreactive area tended to decrease with age. The result was expressed as the c-Kit positive percentage of the total area of each region. Each bar represents the mean ± SE. SMB, submucosal border; MP, myenteric plexus; CM, circular muscle; LM, longitudinal muscle.*P < 0.05 compared with 6-wk-old rats; †*P* < 0.05 compared with 31-wk-old rats; ‡*P* < 0.05 compared with 74-wk-old rats.

The c-Kit-positive area decreased with age (Fig 4A and 4B). In detail, the proportion of the c-Kit-positive area in the SMB of 31-wk-, 74-wk-, and 2-yr-old rats significantly decreased compared to 6-wk-old rats (*P* = 0.027 *vs*. 31-wk-old rats; *P* = 0.018 *vs*. 74-wk-old rats and *P* = 0.036 *vs*. 2-yr-old rats) (Fig 4B). Similarly, in the MP layer, the proportion of the c-Kit-positive area of 74-wk- and 2-yr-old rats significantly was lower than that of the younger age group (*P* = 0.002, 6-wk- *vs*. 74-wk-old rats; *P* < 0.001, 6-wk- *vs*. 2-yr-old rats and *P* = 0.002, 31-wk- *vs*. 2-yr-old rats) (Fig 4B). Additionally, the total proportion of the c-Kit-positive area in the 2-yr-old rats was lower than that of the 6-, 31-, and 74-wk-old rats in all areas (*P* < 0.001 *vs*. 6-wk-old rats; *P* = 0.037 *vs*. 31-wk-old rats and *P* = 0.002 *vs*. 74-wk-old rats) (Table 1).

**Table 1:** The proportion of the c-Kit positive area in the corpus of four different aged rat stomach (all, *n* = 6).

	6 week	31 week	74 week	2 year	*P*-value
**The proportion of c-Kit in SMB (%)**	1.65 ± 1.26	0.9 ± 0.78	0.80 ± 0.51	0.83 ± 0.50	0.008
**The proportion of c-Kit in CM (%)**	0.14 ± 0.09	0.19 ± 0.15	0.19 ± 0.10	0.18 ± 0.11	0.524
**The proportion of c-Kit in MP (%)**	2.04 ± 1.51	1.55 ± 0.93	0.83 ± 0.49	0.30 ± 0.25	< 0.001
**The proportion of c-Kit in LM (%)**	0.15 ± 0.12	0.23 ± 0.15	0.33 ± 0.24	0.17 ± 0.16	0.054
**Total density of c-Kit (%)**	1.05 ± 1.31	0.70 ± 0.81	0.54 ± 0.46	0.37 ± 0.40	< 0.001

The data are expressed as mean ± SD or *n* (%), as appropriate. The groups were compared in terms of ANOVA with post-hoc Tukey HSD in different aged rat group. SMB, submucosal border; CM, circular muscle; MP, myenteric plexus; LM, longitudinal muscle.

### Decrease of *c-Kit*, *SCF and HO-1* mRNA expression with aging

**Fig 5:**
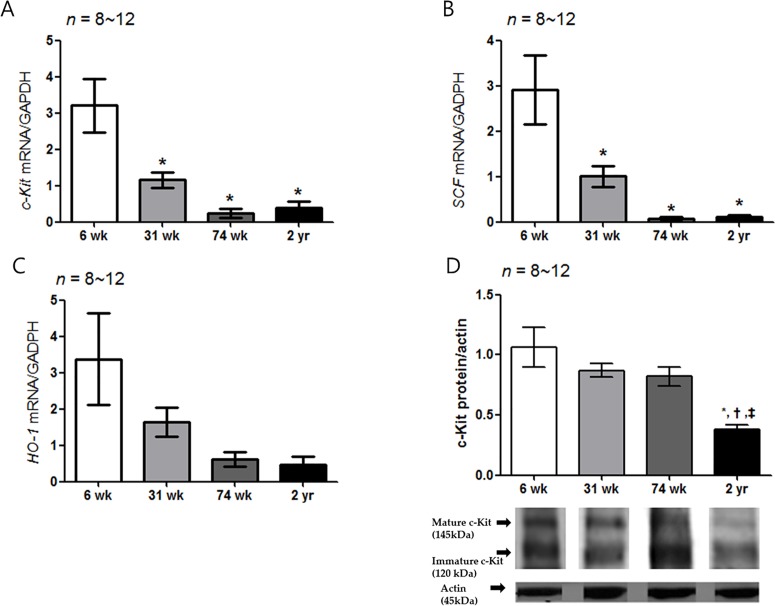
Expression of *c-Kit*, *SCF*, *HO-1* mRNA and c-Kit protein. (A) The expression of *c-Kit* mRNA by real-time PCR decreased with age. The *c-Kit* mRNA expression decreased significantly in the 31-, 74-wk-old and 2-yr-old rats compared with the 6-wk-old rats (*P* = 0.002 *vs*. 31-wk-old rats and *P* < 0.001 *vs*. 74-wk- and 2-yr-old rats): 6-wk-old rat group (*n* = 10), 31-wk-old rat group (*n* = 10), 74-wk-old rat group (*n* = 8) and 2-yr-old rat group (*n* = 12). (B) The *SCF* mRNA expression of other aged rat group was significantly lower than that of the 6-wk-old rat (*P* = 0.004 *vs*. 31-wk-old rats; *P* < 0.001 *vs*. 74-wk- and 2-yr-old rats) (*n* = 8–12). (C) No statistical difference was showed the *HO-1* mRNA expression in different age rat group. (D) The c-Kit protein expression decreased with age. The c-Kit protein expression of 2-yr-old rats was lower than that of the other aged rat groups (*P* < 0.001 *vs*. 31-wk-old rats; *P* = 0.001 *vs*. 74-wk-old rats and *P* = 0.009 *vs*. 2-yr-old rats) (*n* = 8–12). The results are shown as the mean value of the optical density (OD). The optical densities corresponding to the mature (145 kDa) and immature (120 kDa) forms were combined in the analysis process using densitometry. Each bar represents the mean ± SE. *P < 0.05 compared with 6-wk-old rats. †*P* < 0.05 compared with 31-wk-old rats; ‡*P* < 0.05 compared with 74-wk-old rats.

The *c-Kit* mRNA expression decreased significantly in the 31-, 74-wk-old and 2-yr-old rats compared with the 6-wk-old rats (*P* = 0.002 *vs*. 31-wk-old rats and *P* < 0.001 *vs*. 74-wk- and 2-yr-old rats) (Fig 5A). Similarly, the *SCF* mRNA expression of other aged rat group was significantly lower than that of the 6-wk-old rats, respectively (*P* = 0.004 *vs*. 31-wk-old rats and *P* < 0.001 *vs*. 74-wk- and 2-yr-old rats) (Fig 5B). The *HO-1* mRNA expression did not show statistically significant difference in rats of different age group (Fig 5C). The c-Kit protein expression of the 2-yr-old rats was significantly lower than those of the other age groups (*P* < 0.001 *vs*. 31-wk-old rats; *P* = 0.001 *vs*. 74-wk-old rats and *P* = 0.009 *vs*. 2-yr-old rats) (Fig 5D and S1 Fig), respectively.

### Influence of aging on the nNOS-density in the rat corpus

**Fig 6:**
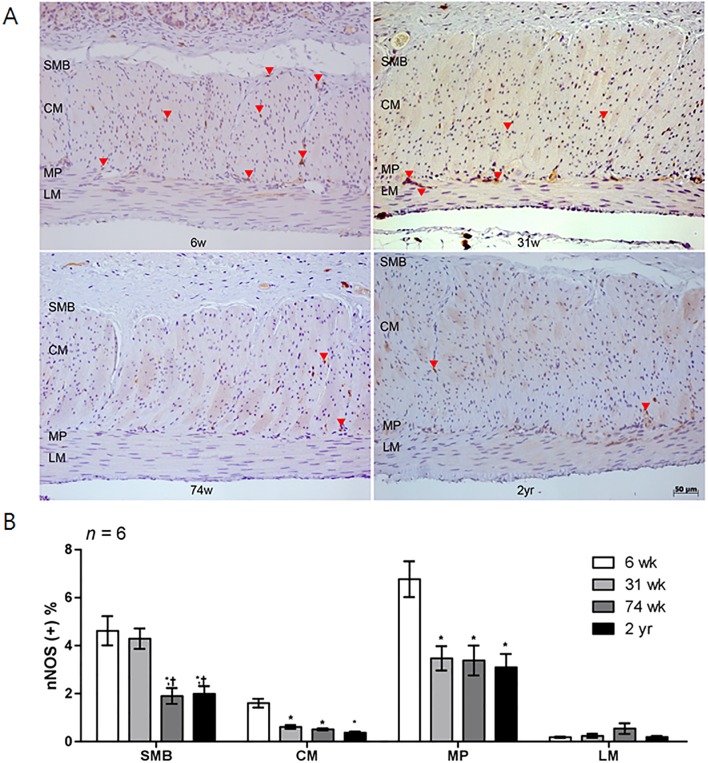
Analysis of nNOS immunohistochemistry. (A) Photomicrograph of nNOS immunostaining of the corpus of rat stomach. Arrows and arrowheads indicate the nNOS-positive nerve fibers and neuronal ganglion, respectively (x200 magnification). (B) Comparison of the nNOS positive area (*n* = 6 per group). The proportion of the nNOS immunoreactive area decreased with age. Each bar represents the mean ± SE. SMB, submucosal border; MP, myenteric plexus; CM, circular muscle; LM, longitudinal muscle.*P < 0.05 compared with 6-wk-old rats; †*P* < 0.05 compared with 31-wk-old rats; ‡*P* < 0.05 compared with 74-wk-old rats.

**Fig 7:**
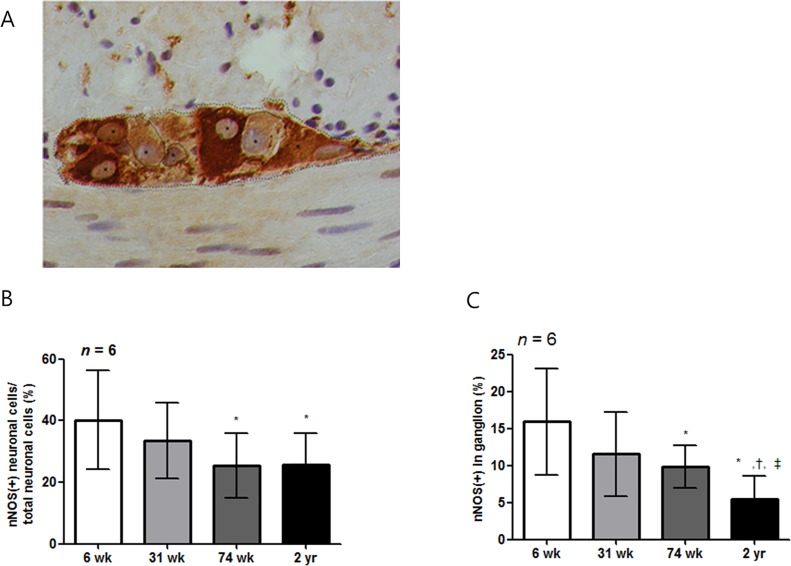
The enumeration of neuronal cell in myenteric ganglia. (A) ganglia in the myenteric plexus were micro graphed at x1,000. We enumerated the nNOS-positive (indicated with red line) or -negative (indicated with black line) myenteric neurons, which include nucleus (indicated with asterisk), in the ganglion (contours of the ganglion are indicated by dashed lines). (B) The percent of nNOS positive neuronal cells of total neuronal cells in ganglion of 74-wk-old and 2-yr-old rats was significantly lower than that of 6- and 31-wk-old rats (*P* = 0.002, 6-wk- *vs*. 74-wk-old rats; *P* = 0.002, 6-wk- *vs*. 2-yr-old rats; *P* = 0.041, 31-wk- *vs*. 74-wk-old rats, and *P* = 0.019, 31-wk- vs 2-yr-old rats). (C) Mean of proportion of nNOS-immunoreactive area in ganglion was also decreased as age increased (*P* = 0.020, 6-wk- *vs*. 31-wk-old rats; *P* = 0.001, 6-wk- *vs*. 74-wk-old rats; *P* < 0.001, 6-wk- *vs*. 2-yr-old rats; *P* < 0.001, 31-wk- *vs*. 2-yr-old rats, and *P* < 0.001, 74-wk- vs 2-yr-old rats). Each bar represents the mean±SE. *P < 0.05 compared with 6 wk of age; †*P <* 0.05 compared with 31-wk of age; ‡ *P* < 0.05 compared with 74-wk-old rats.

**Fig 8:**
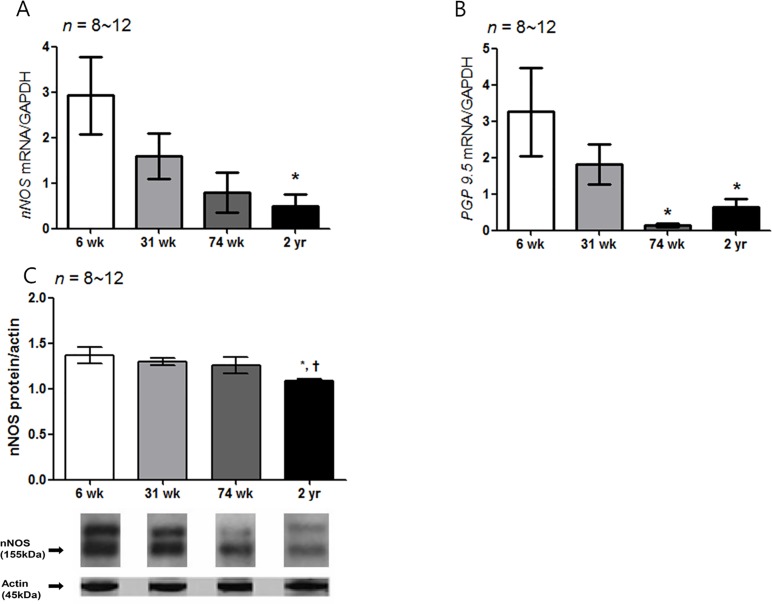
Expression of *nNOS* and *PGP 9*.*5* mRNA and nNOS protein. (A) The expression of *nNOS* mRNA measured by real-time PCR decreased with age. The *nNOS* mRNA expression decreased in the 2-yr-old rats compared with the 6- wk-old rats (*P* = 0.021): 6-wk-old rat group (*n* = 10), 31-wk-old rat group (*n* = 10), 74-wk-old rat group (*n* = 8) and 2-yr-old rat group (*n* = 12). (B) The *PGP 9*.*5* mRNA expression of the 74-wk-old and 2-yr-old rats was also lower than that of the 6-wk-old rats (*P* = 0.007, 6-wk- *vs*. 74-wk-old rats and *P* = 0.030, 6-wk- *vs*. 2-yr-old rats), respectively. (C) The nNOS protein expression was the lowest in 2-yr-old rats. That is, the nNOS protein expression of the 2-yr-old rats was significantly lower than that of the 6-wk-old rats (*P* = 0.007) (*n* = 8–12 in 6-, 31-, 74-wk- and 2-yr-old rats). The results are shown as the mean value of the optical density (OD). Each bar represents the mean ± SE. *P < 0.05 compared with 6-wk-old rats; †*P* < 0.05 compared with 31-wk-old rats; ‡*P* < 0.05 compared with 74-wk-old rats.

Similar to c-Kit IHC, a larger proportion of nNOS-positive area was present in the 6-wk-old rats, and it decreased with age in the four layers of the gastric stomach (Table 2 and Fig 6A). In the SMB, the proportion of the nNOS-positive area of the 74-wk-old and 2-yr-old rats was lower than that of the 6- and 31-wk-old rats (*P* = 0.001 *vs*. 6-wk-old rats; *P* = 0.003, 31-wk- *vs*. 74-wk-old rats and *P* = 0.007, 31-wk- *vs*. 2-yr-old rats) (Fig 6B). The nNOS-positive area in CM and MP layer was decreased in aged rat groups compared with 6-wk-old rats (*P* < 0.001 *vs*. all other aged rat group in CM layer; *P* = 0.001 *vs*. 31-wk-old rats; *P* = 0.002 *vs*. 74-wk-old rats and *P* = 0.001 *vs*. 2-yr-old rats in MP layer) (Fig 6B). Fig 7A showed the analysis of nNOS neuronal cell in myenteric ganglion. When myenteric ganglion was analyzed, total neuronal cell count did not show statistically significant difference in rats of different age group. However, the percent of nNOS-positive neuronal cell per total neuronal cell of 74-wk- and 2-yr-old rats significantly decreased compared with 6-wk-aged rat (*P* < 0.001) (Fig 7B). The proportion of nNOS-immunoreactive area of 2-yr-old rats were significantly lower than that of 6-, 31-, and 74-wk-old rats (*P* < 0.001 *vs*. 6-wk-old rats; *P* = 0.002, *vs*. 31-wk-old rats and *P* = 0.017, vs. 74-wk-old rats) (Fig 7C). Similarly, the *nNOS* mRNA expression decreased in the 2-yr-old rats compared with the 6-wk-old rats (*P* = 0.021) (Fig 8A). The *PGP 9*.*5* mRNA expression of the 74-wk- and 2-yr-old rats was also lower than that of the 6-wk-old rats (*P* = 0.007, 6-wk- *vs*. 74-wk-old rats and *P* = 0.030, 6-wk- *vs*. 2-yr-old rats), respectively (Fig 8B). The nNOS protein expression was the lowest in 2-yr-old rats and the nNOS protein expression of the 2-yr-old rats was significantly lower than that of the 6-wk-old rats (*P* = 0.007) (Fig 8C, S1 Fig).

**Table 2:** The proportion of the nNOS positive area in the corpus of four different aged rat stomach (all, *n* = 6).

	6 week	31 week	74 week	2 year	*P*-value
**The proportion of nNOS in SMB (%)**	4.62 ± 3.04	4.29 ± 2.19	1.90 ± 1.43	1.99 ± 1.40	<0.001
**The proportion of nNOS in CM (%)**	1.60 ± 0.92	0.61 ± 0.41	0.51 ± 0.18	0.37 ± 0.22	<0.001
**The proportion of nNOS in MP (%)**	6.77 ±3.74	3.47 ± 2.59	3.38 ± 2.72	3.10 ± 2.41	<0.001
**The proportion of nNOS in LM (%)**	0.18 ± 0.16	0.24 ± 0.18	0.54 ± 0.48	0.19 ± 0.16	0.101
**Total density of nNOS (%)**	3.29 ± 3.56	2.12 ± 2.44	1.56 ± 1.97	1.41 ± 1.85	<0.001

The data are expressed as mean ± SD or *n* (%), as appropriate. The groups were compared in terms of ANOVA with post-hoc Tukey HSD in different aged rat group. SMB, submucosal border; CM, circular muscle; MP, myenteric plexus; LM, longitudinal muscle.

### Change in gastric accommodation with age

**Fig 9:**
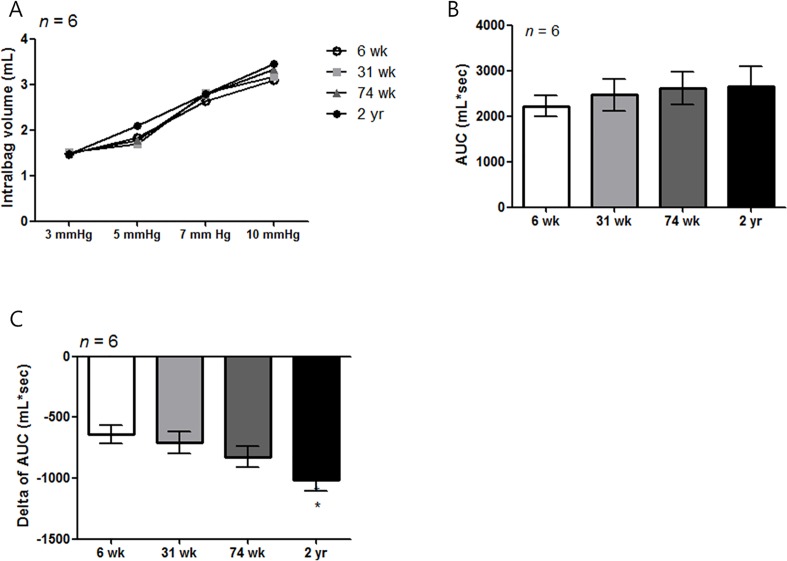
Measurement of gastric volume change and influences of L-NAME in different aged rat group. (A) The graph of pressure dependent volume curve did not show statistical difference in each given pressure (*n* = 6 per group). (B) Pressure dependent AUC (mL*sec) curve did not show any difference as aging (*n* = 6 per group). After treatment with L-NAME (10 mg/kg), the delta of the AUC significantly decreased in the 2-yr-old rats compared with the 6-wk-old age rats (*P* < 0.05). *P < 0.05 compared with 6-wk-old rats.

Among the four different age groups (all, n = 6), there was no statistically significant difference in the pressure dependent volume change (Fig 9A) and AUC (mL*sec) of the pressure-dependent volume curve in tonic phase (Fig 9B). After the administration of L-NAME, ΔAUC in the 2-yr-old rat group (- 1072.6 ± 81.3 mL*sec) decreased significantly compared with the 6-wk-old rat group (- 678.0 ± 180.79 mL*sec) (*P* = 0.003) (Fig 9C).

## Discussion

The present study showed a decrease in the immunoreactive density and molecular expression in ICC and in nNOS in the aged F344 rat stomach (corpus area) but our barostat experiment did not show decrease of gastric relaxation in the old rats. Instead, gastric relaxation was significantly decreased in the presence of L-NAME in the 2-yr aged rat group, supporting the role of nNOS in the relaxation of gastric stomach in the old rat.

The changes in gastrointestinal tract motility that occur with advanced age may reflect a selective loss or selective impairment of function involving a subpopulation of neurons [11]. Similarly, the effect of aging on gastric motility has gained attention because functional dyspepsia, such as postprandial discomfort syndrome, has been reported to be common in the aged population [39, 40]. A previous study reported that aging caused a decrease in gastric motility, such as a decline in the compliance of the stomach [11], and its role in the anorexia of aging has been discussed together with antral distension and delayed emptying [2]. However, Philipps et al reported that age-related cell loss in the myenteric plexus does not occur in nitrergic neurons; instead, it occurs exclusively in the cholinergic subpopulation of enteric neurons in Fischer 344 rats [11, 14]. Previously, a definite age-associated decline of the nNOS levels has been reported in gastric mucosa [16] as well as in colon muscle [16, 17, 41]. The decrease in nNOS-immunoreactive neuronal cells which are associated with age might also be related to the damage of nitrergic neurons, such as axonal swelling and the loss of expression of NOS in aged rats [14, 42]. Likewise, we found the decrease of both nNOS-positive neuronal cells per total neuronal cells and the proportion of nNOS-immunoreactive area in the corpus of 2-yr-old-rats in the present study. Decreased ΔAUC means that the relaxation was more inhibited after given L-NAME. Thus, the decrease of nNOS in the aged rat might cause less gastric relaxation at the same dose of L-NAME in comparison to the young aged rat group. Disappointingly, pressure dependent volume curve and AUC did not show proportional change depending on age. Instead, relative decrease in the spontaneous gastric relaxation was found after the L-NAME treatment in the 2-yr-old rats. That is, decrease of nNOS neuron in the aged rat group might cause more suppression of gastric relaxation with the same dose of L-NAME treatment resulting in the decreased ΔAUC in the 2-yr-old rat group. Our functional study suggests that some proportion of the gastric relaxation has been mediated by nNOS inhibitory transmission although nNOS is not the main factor. An additional explanation could be that the decreasing ICC number in the aged rat might influence the gastric relaxation by decreasing transfer neurotransmitter action from neuronal cells. Another possibility is that NO action can be activated more by decrease of ICC cell number in the aged rat. However, these explanations need further experiments. It is well known that the NO is the predominant factor of gastric relaxation [43, 44], and gastric relaxation is regulated by activation of the cyclic guanosine monophosphate (cGMP)-dependent mechanisms [43, 45, 46]. Based on previous studies, ICC is known to be a significant component in the regulation of normal gastrointestinal fuctioning [47–49]. Indeed, several human gastrointestinal motility disorders have been associated with ICC depletion [49]. Previously, immunohistochemistry showed Kit adjacently located to the M3 receptor revealed their role in excitatory neurotransmission [50]. In contrast, NO-sensitive guanylyl cyclase (NO-GC) was found to be expressed in ICC [51–53] and this this expression of NO-GC in ICC was stronger than in surrounding smooth muscle cell (SMC) which revealed the role of ICC in nitrergic inhibitory signaling [51, 52]. However, there is still an unresolved controversy of the primary targets of NO (whether SMC or ICC). Despite the previous *in vitro* evidence for ICC role in neurotransmission, a study was done on isolated whole stomach of wild-type and W/W^v^ mice which found that normal gastric distension-induced adaptive relaxation occurred in both mice groups [54].

In another animal study using manometry [55], the lower esophageal sphincter (LOS) in the nNOS^-/-^ mice was elevated and relaxation was decreased compared with W/W^v^ mice [56]. Previously, Burns et al reported that the reduced NO-dependent inhibitory neuroregulation caused relaxation of smooth muscle tissues in response to exogenous sodium nitroprusside, which means that ICC-IM have an important role in NO-dependent neurotransmission in the stomach [57]. These results negate the role for ICC in neural transmission. *In vitro* experiment using circular smooth muscle tissue from LOS of wild type and W/W^v^ mutant mice [58], this study suggested that significant variability was found in the generation of nitrergic neurotransmission in the LOS of W/W^v^ mutant mice, whereas purinergic and cholinergic neurotransmissions are intact. The purinergic and cholinergic neurotransmissions had altered nitrergic responses that appear to be associated with abnormal Ca^2+^-dependent signaling which was initiated by spontaneous Ca^2+^ release from sarcoplasmic reticulum in smooth muscle cells, and so c-Kit-positive ICC are not essential for nitrergic neurotransmission in mouse LOS smooth muscle [58]. Taken together with the present results, it seems that the decreased ICC with aging did not directly affect the relaxation in the stomach. Furthermore, it was found the inhibitory effect of aging on the rat gastric fundus relaxation response is mainly mediated by the nitrergic pathway.

This present study had several limitations. First, we measured the change of nNOS as the proportion of immunoreactive area (% of total area). This method might be valid as a generic indication of changes of neuron. Overall, the way to visualize immunoreactivity is not the most appropriate for quantitation because optical density is less reliable than fluorescence to detect areas of immunoreactivity. Second, we used corpus (proximal glandular stomach) area of rat stomach for the quantitative (morphological) or molecular experiment instead of forestomach. Although many previous studies [22, 38, 44, 59] reported about the experiment of gastric relaxation in murine, there was no definite mention about the precise region of fundus in the murine stomach. The stomach of the rat is divided into the forestomach (pars proventricularis) and glandular stomach (corpus or pars glandularis), which is different from human. Some scientists do not believe the forestomach has muscle as it looks pale and relatively thin. Thus, if the reports do not show the experiment region as a figure as the present study there could be some confusion which part of stomach was analyzed for gastric accommodation experiments. The corpus of rat stomach might not be ideal for comparative analyses for gastric relaxation due to its variability and small contribution to volume changes. In the preliminary study, we found that the layer of forestomach was rather thin and less expansible, not feasible for reflecting the change of gastric relaxation as aging. That is the reason why we selected the area of rat corpus for gastric relaxation experiment in the present study. In addition, gastric accommodation not only involves the proximal part of the stomach, but also the distal part of the stomach by the presence of the antrofundic reflex. Using this corpus area, our present study clearly has shown the age-related changes in the density and molecular expression of ICC and nNOS-positive enteric neurons in the muscle layer of stomach in the four different ages as well as IHC study in the whole area. However, we could not reveal the decreased gastric relaxation associated with aging in the present study. The baseline pressure dependent volume curve did not show any differences among different aged rat groups. Generally, it would be ideal to compare the change of gastric volume before and after meal for the measurement of gastric accommodation in the different aged rat groups. However, our method of balloon insertion status via mouth to stomach made it very difficult to measure gastric relaxation after meals. That is, the rat that suddenly died after ingesting a meal probably died giving the meal ingestion probably due to asphyxia with technical error. After repeated struggle we decided to quit the barostat experiment after eating. Ideally the barostat experiment for evaluation of gastric relaxation should be performed in the different aged human model but it was found in this study to be difficult. Thus we proposed a new setting in the murine for gastric accommodation model in the fasting status, which might not be sufficient. As far as our knowledge, this is the first study which has evaluated the change of gastric relaxation using a functional barostat machine in the different ages of rat model. Furthermore, the response to L-NAME treatment showed a significant change in the 2-yr-old rat group. It could be a similar phenomenon in our previous report in which the proximal colon contractile response to EFS under L-NAME was significantly larger in proximal colon of aged rat [16]. This decreased response to L-NAME in the aged rats might reflect the degeneration of nitrergic neurons. Furthermore, there are many varying factors relating relaxation in GI muscles, except NO, such as choline acetyltransferase, dopamine, VIP related peptide, ATP and 5-HT. Moreover, the association between NO and other neurotransmitter in gastric relaxation mechanism was not fully eludicidated, and our present functional study could present the limited role of NO in gastric relaxation as aging. Thus, the further experiments are needed using other excitatory and inhibitory neurotransmitters. In conclusion, the morphologic and molecular nNOS changes but not ICC in the stomach might play a role in the decrease of gastric accommodation with age.

## Supporting Information

S1 Fig The Western blot analysis of c-Kit in different aged rat group. The original data of c-Kit protein analysis in different aged rat groups; 6-wk-old rat group (*n* = 10), 31-wk-old rat group (*n* = 10), 74-wk-old rat group (*n* = 8) and 2-yr-old rat group (*n* = 12).
(TIF)

S2 Fig The Western blot analysis of nNOS in different aged rat group. The original data of nNOS protein analysis in different aged rat groups; 6-wk-old rat group (*n* = 10), 31-wk-old rat group (*n* = 10), 74-wk-old rat group (*n* = 8) and 2-yr-old rat group (*n* = 12).
(TIFF)
