# Correction: Change in the Interstitial Cells of Cajal and nNOS Positive Neuronal Cells with Aging in the Stomach of F344 Rats

**DOI:** 10.1371/journal.pone.0179107

**Published:** 2017-06-01

**Authors:** Yong Hwan Kwon, Nayoung Kim, Ryoung Hee Nam, Ji Hyun Park, Sun Min Lee, Sung Kook Kim, Hye Seung Lee, Yong Sung Kim, Dong Ho Lee

The following information is missing from the Funding section: This work was supported by the National Research Foundation of Korea (NRF) grant for the Global Core Research Center (GCRC) funded by the Korea government (MSIP) (No. 2011–0030001).

In Fig 6, panel A appears incorrectly. Please see the correct Fig 6 here.

**Fig 6:**
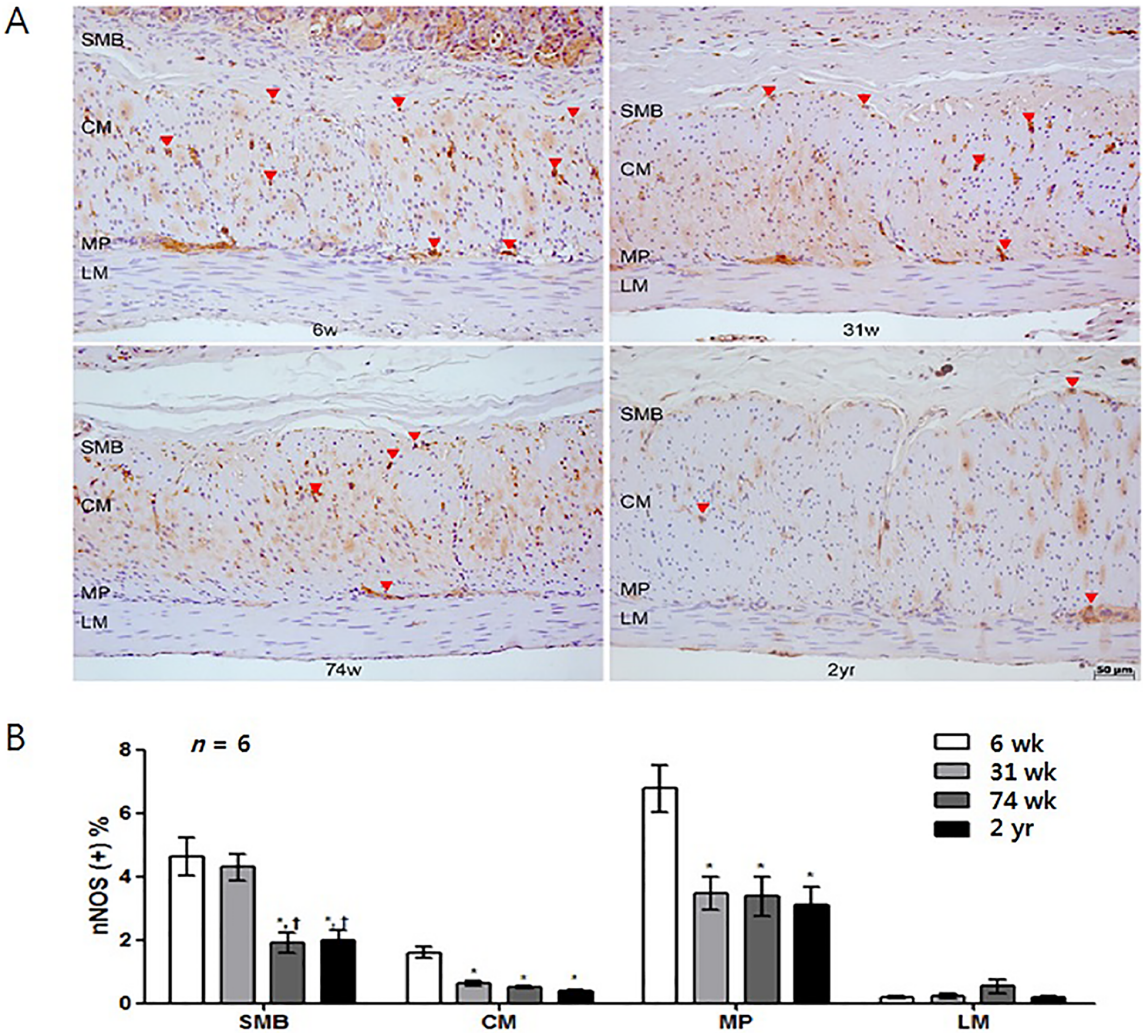
Analysis of nNOS immunohistochemistry. (A) Photomicrograph of nNOS immunostaining of the corpus of rat stomach. Arrows and arrowheads indicate the nNOS-positive nerve fibers and neuronal ganglion, respectively (x200 magnification). (B) Comparison of the nNOS positive area (*n* = 6 per group). The proportion of the nNOS immunoreactive area decreased with age. Each bar represents the mean ± SE. SMB, submucosal border; MP, myenteric plexus; CM, circular muscle; LM, longitudinal muscle.*P < 0.05 compared with 6-wk-old rats; †*P* < 0.05 compared with 31-wk-old rats; ‡*P* < 0.05 compared with 74-wk-old rats.
