# Supplementary figures and images for: Change in the Interstitial Cells of Cajal and nNOS Positive Neuronal Cells with Aging in the Stomach of F344 Rats
Source: PLoS One. 2017 Jan 3;12(1):e0169113. doi: 10.1371/journal.pone.0169113 (PMC5207530; doi:10.1371/journal.pone.0169113)

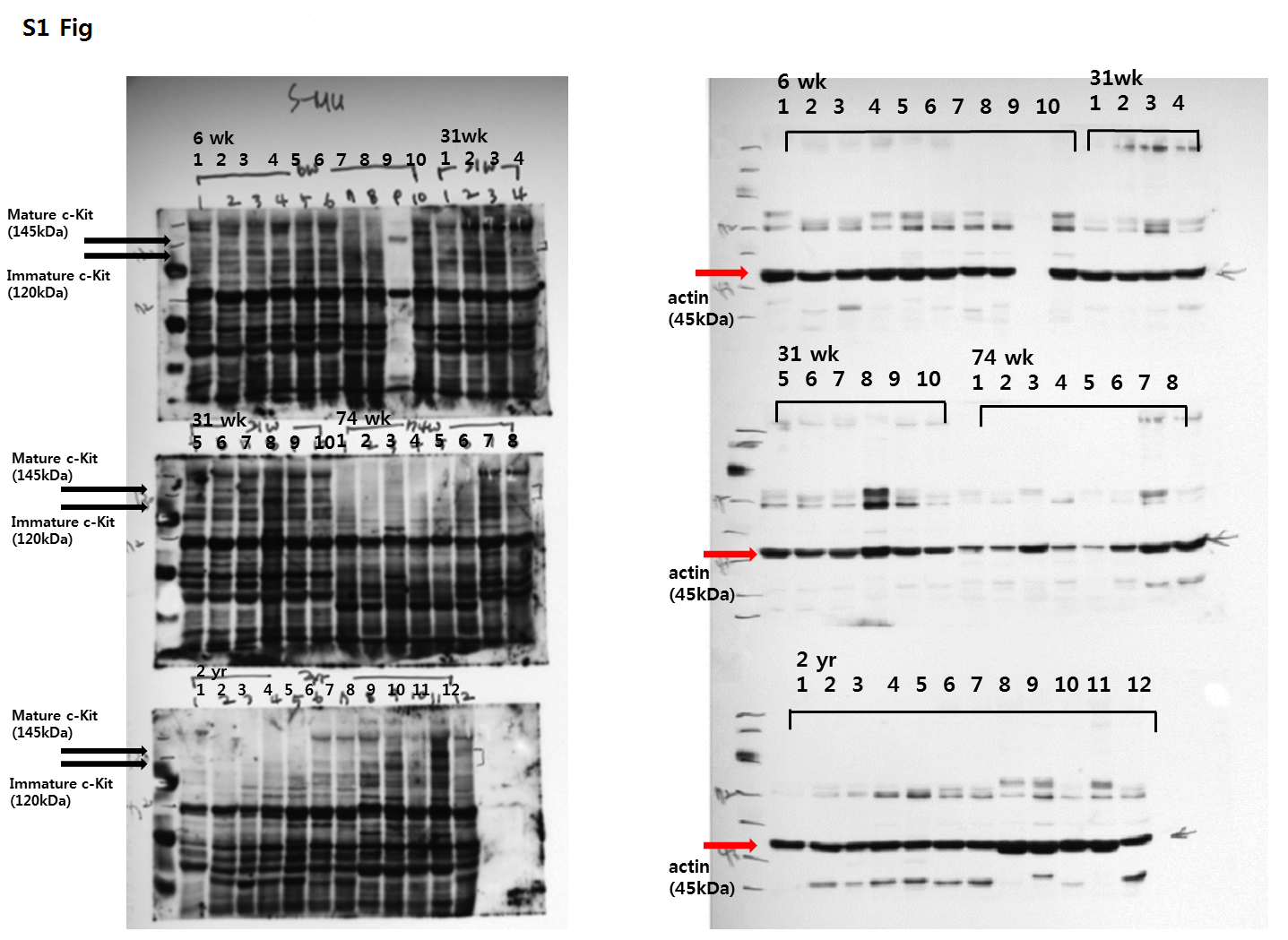

Supplement: S1 Fig — The original data of c-Kit protein analysis in different aged rat groups; 6-wk-old rat group (n = 10), 31-wk-old rat group (n = 10), 74-wk-old rat group (n = 8) and 2-yr-old rat group (n = 12). (TIF) [file pone.0169113.s001.tif]

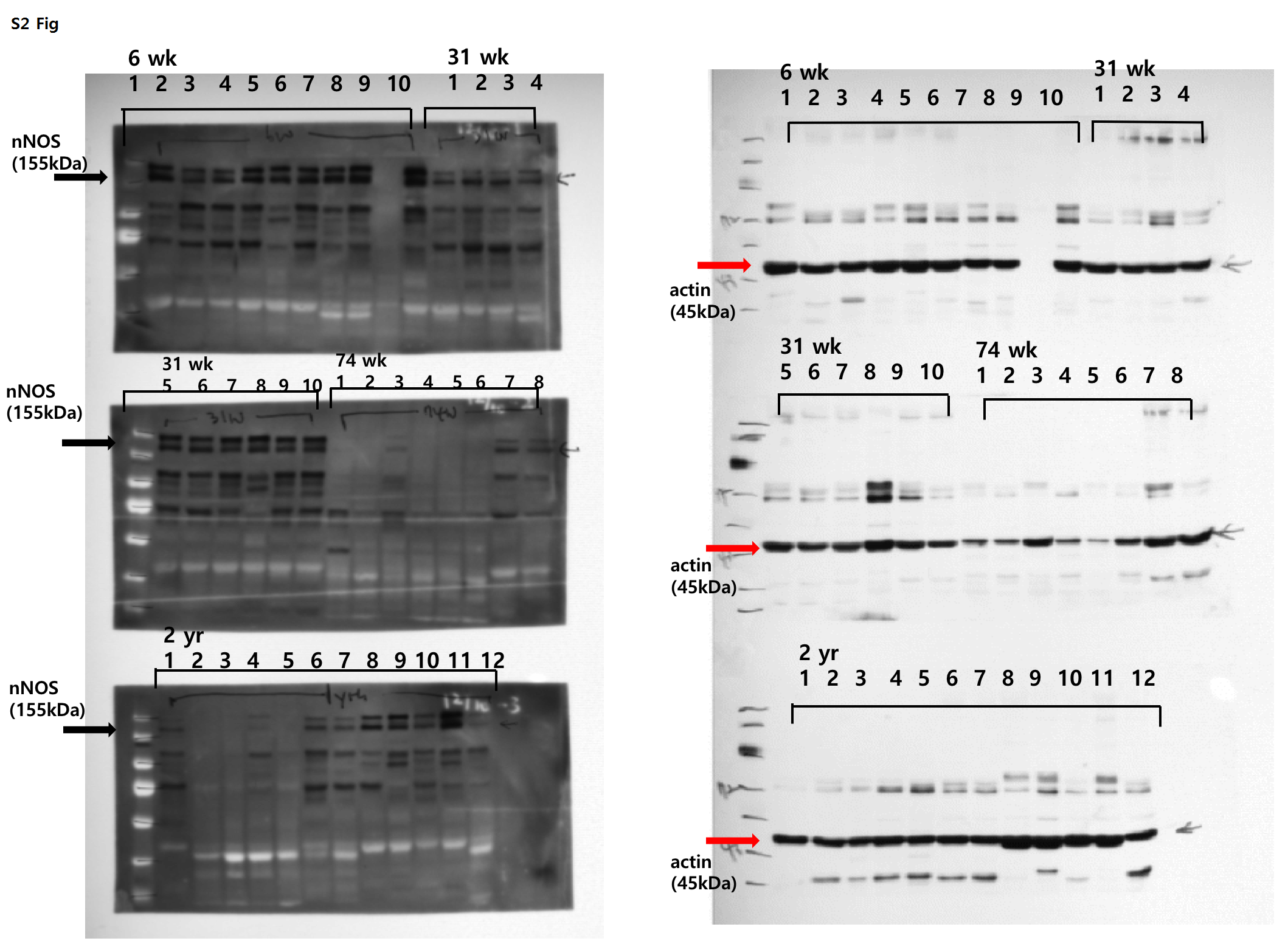

Supplement: S2 Fig — The original data of nNOS protein analysis in different aged rat groups; 6-wk-old rat group (n = 10), 31-wk-old rat group (n = 10), 74-wk-old rat group (n = 8) and 2-yr-old rat group (n = 12). (TIFF) [file pone.0169113.s002.tiff]
